# MOTIVATIONS AND ROLE CAPTIVITY AMONG INFORMAL CAREGIVERS TO COMMUNITY-DWELLING PERSONS WITH DEMENTIA

**DOI:** 10.1093/geroni/igad104.2411

**Published:** 2023-12-21

**Authors:** Rachel Bloom, Francesco Osso, Megan McCarthy, Francesca Falzarano

**Affiliations:** Fordham University, New York City, New York, United States; Weill Cornell Medicine, New York City, New York, United States; Weill Cornell Medicine, New York City, New York, United States; Weill Cornell Medicine, New York City, New York, United States

## Abstract

There are 11 million informal (unpaid) caregivers currently assisting a person with dementia in the United States. Motivations for taking on this challenging role vary, with many citing their responsibility to family or a shared interpersonal history. Many caregivers do not feel as if they have a choice in taking on care responsibilities for relational and/or financial reasons and are held “captive” in this role. Prior research indicates role captivity as a key mediating force in the development of depression among dementia caregivers, but limited work has directly linked quantitative measures of role captivity with qualitative data surrounding motivations for providing care. The current mixed-methods study uses quantitative and qualitative data to explore this connection in a sample of 46 participants providing care to a community-dwelling person with dementia. Study findings show that role captivity was significantly associated with greater costs of care, feelings of grief, and depressive symptoms, as well as lower levels of self-efficacy and mutuality. These relationships were assessed alongside thematic analyses of interview data centered on motivations for providing care, familial and cultural expectations, and financial necessity. This research paints a detailed picture of the way in which dementia caregivers understand and relate to their role and can inform the design of effective psychological supports for this population.

